# Supplementary figures and images for: Heme-induced genes facilitate endosymbiont (Sodalis glossinidius) colonization of the tsetse fly (Glossina morsitans) midgut
Source: PLoS Negl Trop Dis. 2022 Nov 28;16(11):e0010833. doi: 10.1371/journal.pntd.0010833 (PMC9731421; doi:10.1371/journal.pntd.0010833)

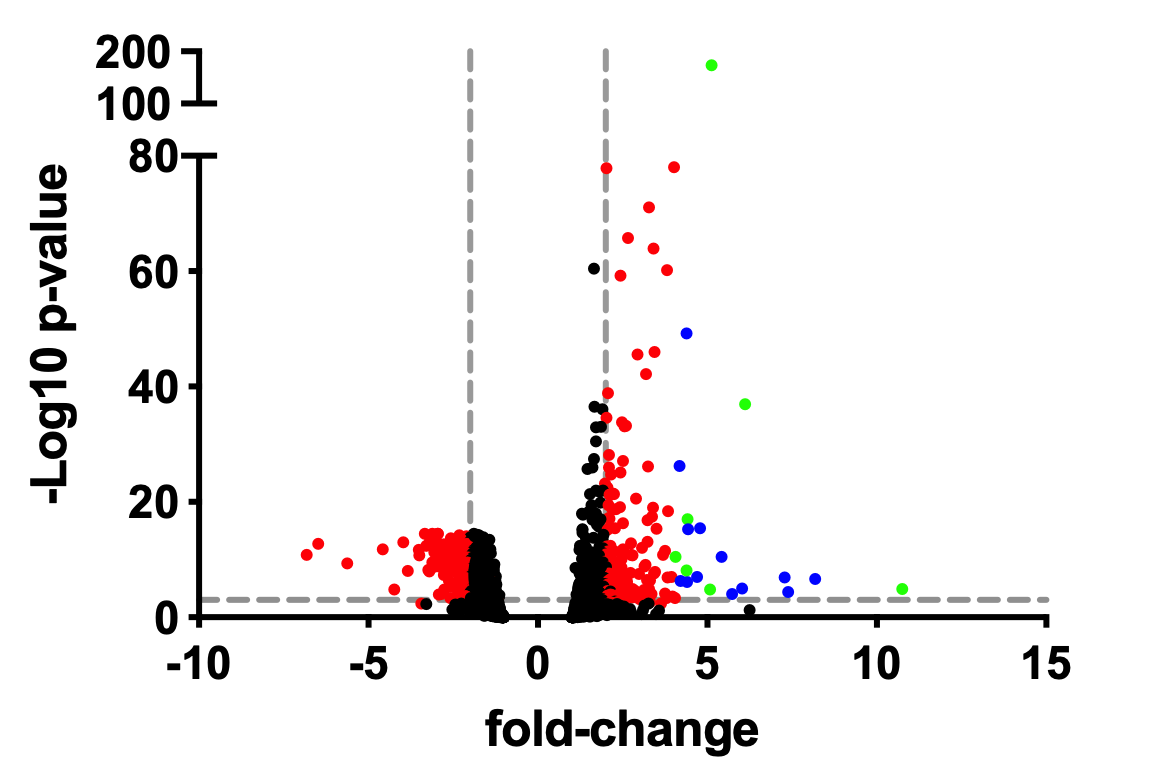

Supplement: S1 Fig — All Sgm genes detected by RNA-seq are plotted on the graph, and each dot represents one gene. Genes represented in red are significantly differentially expressed (p-value of ≤ 0.05 and a fold-change ≥ 2, indicated by dashed, grey lines) in treatment vs. control cells. Green and blue genes are listed in Table 2, and green genes represent those experimentally mutated in Sgm and assayed for their colonization phenotype in tsetse’s gut (results shown in Fig 4). (TIF) [file pntd.0010833.s001.tif]
