# Supplementary material for: Heme-induced genes facilitate endosymbiont (Sodalis glossinidius) colonization of the tsetse fly (Glossina morsitans) midgut
Source: PLoS Negl Trop Dis. 2022 Nov 28;16(11):e0010833. doi: 10.1371/journal.pntd.0010833 (PMC9731421; doi:10.1371/journal.pntd.0010833)
Supplement: S1 Table — (DOCX) [file pntd.0010833.s002.docx]

**S1 Table. PCR primers used in this study.**

| **Primer pairs** | **Sequence** | **DNA amplified** |
| --- | --- | --- |
| SodrplB1F SodrplB1R | 5’ TGCTGGAAACTCTCAGCAAAT | *Sodalis rplB* |
| SodrplB1R | 5’ CTCCAGACGTTCTACCACTGC |  |
|  |  |  |
| UR285 | 5’ CTAGTCTAGACTTCTCATTCATGCAGCAACA | *Sodalis* SG1505^a^ |
| UR286 | 5’CCGCTCGAGATTATCTGAGCAGCCGCAATA |  |
|  |  |  |
| UR606 | 5’ CTGGTCGAAAAAGCCGTATC | *Sodalis* SG0074^a^ |
| UR607 | 5’ CCGCTCAGTGTTTCGGTAAT |  |
|  |  |  |
| UR566 | 5’ GCCGGCATAGATATCGGTAA | *Sodalis* SG2427^a^ |
| UR567 | 5’ CATTTCACATGGTCGCTTTG |  |
|  |  |  |
| UR608 | 5’ ACGCTGGTCACCGATAAAAC | *Sodalis* SG2179^a^ |
| UR609 | 5’ TTCCGGCGTAAGCAAGTTAT |  |
|  |  |  |
| UR590 | 5’ CAAAACATCGATGGCAACAC | *Sodalis* SG2061^a^ |
| UR591 | 5’ ACCGTGCTTTGGTTCTCTTG |  |
|  |  |  |
| UR616 | 5’ AGGTGGCATTGGTTCTCCTG | *Sodalis* SGP2_0009^a^ verification) |
| UR617 | 5’ AAGCAGCTCCTCCCATTTCA |  |
|  |  |  |
| UR627 | 5’ CGACTGGCAGAACTGACGAT | *Sodalis* SGP2_0002^a^ |
| UR628 | 5’ CAGCTCCCAGCTTTTCATGC |  |
|  |  |  |
| UR629 | 5’ GGCTGTTTTTGATCTGGCGG | *Sodalis* SG0437^a^ |
| UR630 | 5’ GCAGGTTGTTTTTGTCGGTGA |  |
|  |  |  |
| UR631 | 5’ GGTTGACCACCTGTGTAGCC | *Sodalis* SG1100^a^ |
| UR632 | 5’ CGGTTGCGAGGGATGTAGAA |  |
|  |  |  |
| UR659 | CCGGTCCCCGGAACAATAAT | *Sodalis* SG0182^a^ |
| UR660 | GAGTTGAAGGTTTGCGCCAG |  |
|  |  |  |
| UR681 | 5’ TCAACAAGCTGCTTGGCAAC | *Sodalis* SG2280^a^ |
| UR682 | 5’ AGCCCCTCCAGGAAGAGAAT |  |
|  |  |  |
| UR679 | 5’ TGAGCGCTTGGTGTAATGACA | *Sodalis* SG1275^a^ |
| UR680 | 5’ GTTCGGCGACATACCATTGC |  |
|  |  |  |
| SG0437F | 5’ CAGGAGATGGGCTATCAGGA | *Sodalis* SG0473^b^ |
| SG0437R | 3’ TACTTCAGCGTGCAATCCAG |  |
|  |  |  |
| SG2427F | 5’GCACCCACCCATAGCATTAT | *Sodalis* SG2427^b^ |
| SG2427R | 5’ATTGAAGCCGAGATGTACCG |  |
|  |  |  |
| SGP2_0009F | 5’ GGTGAAGAGGCAGAAGATGC | *Sodalis* SGP2_0009^b^ |
| SGP2_0009R | 5’ GTTGATGTTGACACCATGGG |  |
|  |  |  |
| SG1100F | 5’ CGCCAAAGGTTTTGGTTTTA | *Sodalis* SG1100^b^ |
| SG1100R | 5’ CAACAGTCTCGGCTTCAACA |  |
|  |  |  |
| SPG1_0002F | 5’ TGAAGGTGAAGGGTTTACCG | *Sodalis* SPG1_0002^b^ |
| SPG1_0002R | 5’ CCTTTGCCTGTCCATGTTTT |  |
|  |  |  |
| SG1275F | 5’ GCAGATGATCACGCTGAAGA | *Sodalis* SG1275^b^ |
| SG1275R | 5’ CAGCTTGTCGAGGATGGATT |  |
|  |  |  |
| SG0074F | 5’ ATCTCGGCGATATGCAAAAG | *Sodalis* SG0074^b^ |
| SG0074R | 5’ CCGCTCAGTGTTTCGGTAAT |  |
|  |  |  |
| SG0182F | 5’ GCTGCTGATTCTGCTTACCC | *Sodalis* SG0182^b^ |
| SG0182R | 5’ AGCGGATGAGATAGCTCCAG |  |
|  |  |  |
| SG2179F | 5’ ACGCTGGTCACCGATAAAAC | *Sodalis* SG2179^b^ |
| SG2179R | 5’ TAACTTGCTTACGCCGGAA |  |
|  |  |  |
| SG2061F | 5’ CAAAACATCGATGGCAACAC | *Sodalis* SG2061^b^ |
| SG2061R | 5’ AAGAGAACCAAAGCACGGTC |  |
|  |  |  |
| SG2280F | 5’ TCACGAATCCATTGACGAAA | *Sodalis* SG2280^b^ |
| SG2280R | 5’ GCCCTGCAATTCCAAATAGA |  |
|  |  |  |
| SG1505F | 5’ ACAACCTGGCCTATCAGGTG | *Sodalis* SG1505^b^ |
| SG1505R | 5’ TGTACAGGGCCGGTAAAGAC |  |
|  |  |  |
| SG1621F | 5’ GCTTATGTCGGCAGTGATGA | *Sodalis* SG1621^b^ |
| SG1621R | 5’AGACCAGATGCGACAACAGA |  |
|  |  |  |

^a^Primers used for verification of mutations in genetically engineered *Sgm*.
^b^Primers used for RT-qPCR based analysis of heme regulated genes in *Sgm* that reside in tsetse’s gut.
