# Supplementary material for: Heme-induced genes facilitate endosymbiont (Sodalis glossinidius) colonization of the tsetse fly (Glossina morsitans) midgut
Source: PLoS Negl Trop Dis. 2022 Nov 28;16(11):e0010833. doi: 10.1371/journal.pntd.0010833 (PMC9731421; doi:10.1371/journal.pntd.0010833)
Supplement: S3 Table — (DOCX) [file pntd.0010833.s004.docx]

**S3 Table. Phenotypes associated with selected *Sgm* heme-induced genes.**

| Gene | COG category | Mutant | Putative Function | Tsetse colonization^a^ |
| --- | --- | --- | --- | --- |
| SG1505 | P | URSOD7 | Heme binding and/or degradation | + |
| SG0074 | T | URSOD25 | Universal stress protein A | + |
| SG2427 | G | URSOD26 | PTS system IIA component (for sugars) | - |
| SG2179 | O | URSOD27 | CpxP (regulation of periplasmic stress) | + |
| SG2061 | K,N | URSOD28 | Negative regulator of flagellin synthesis | + |
| SGP1_0002 | K | URSOD31 | DNA binding protein | +/- |
| SG0437 | E | URSOD32 | 2-isopropylmalate synthase (leucine biosynthesis) | + |
| SG1100 | K | URSOD33 | Cold shock like protein | + |
| SG0182 | G | URSOD35 | Fucose transport protein | +/- |
| SG2280 | P | URSOD40 | Bacterioferritin | +/- |
| SG1275 | P | URSOD41 | Ferritin-like protein | + |
| SG1621^b^ | H | N/A | erythronate-4-phosphate dehydrogenase | N/A |

^a^+ indicates that *Sgm* strains with a mutation in the corresponding gene are able to colonize tsetse’s gut at densities similar to that of parent strain *Sgm*^F-PAR^, - indicates that *Sgm* strains with a mutation in the corresponding gene are unable to colonize the tsetse’s gut, +/- indicates that *Sgm* strains with a mutation in the corresponding gene are able to colonize the tsetse’s gut but at a significantly lower density than that of parent strain *Sgm*^F-PAR^.

^b^SG1621 is a heme-repressed gene (HRG), and as such we did not make a corresponding mutant strain. This gene was selected to confirm that an HRG identified *in vitro* was similarly repressed *in vivo*.
