# Supplementary material for: Heme-induced genes facilitate endosymbiont (Sodalis glossinidius) colonization of the tsetse fly (Glossina morsitans) midgut
Source: PLoS Negl Trop Dis. 2022 Nov 28;16(11):e0010833. doi: 10.1371/journal.pntd.0010833 (PMC9731421; doi:10.1371/journal.pntd.0010833)
Supplement: Attachment — Submitted filename: Runyen-Janecky et al_Response to Reveiwers.docx [file pntd.0010833.s005.docx]

8 November, 2022

Dear Dr. Ribeiro, Dr. Picardeau, Dr. Oliveira, and the Anonymous Reviewer,

We would like to thank you very much for your comprehensive review of our manuscript, entitled ‘Heme-induced genes facilitate endosymbiont (*Sodalis glossinidius*) colonization of the tsetse fly (*Glossina morsitans*) midgut’. In the following pages we provide an italicized response to each of the reviewer comments, which are listed in the order they originally appeared. We also provide two versions of the revised manuscript, one with highlighted changes and one ‘clean’ version.

Again, we thank the editors and reviewers for taking the time to read the manuscript and provide valuable feedback about our research, and we hope the manuscript is now suitable for publication in PLoS NTDs.

Sincerely,

Laura Runyen-Janecky, Jack D. Scheutzow, Ruhan Farsin, Leah F. Cabo, Katie E. Wall, Katrina M. Kuhn, Rashel Amador, Shaina J. D’Souza, Aurelien Vigneron, Brian L. Weiss,

Reviewer #1: Figure 2 is not legible.

*We have remade the figure into a more legible format.*

Reviewer #2: About the results I have a couple of specific questions:
Line 279 -figure 3 – as the  labile heme pool in the gut of glossina is not known (see comments on discussion), the term high and low heme is instead a hypothesis than a fact. So a change for something more attached to the experimental condition (just time after a blood meal) would be better.

*As Dr. Oliveira suggested, we have changed the values on the X-axis to reflect the specific times post-feeding at which we measured the expression of each gene.*

Line 396 – if iron is important, then heme degradation should be modulated as well. Are there symbiont heme oxygenase homologs? Or is this ferritin important for handling non-heme iron? Or iron produced by a heme-degrading insect HO?
Is there a functional heme biosynthesis pathway in this bacteria? In the table, there are several genes related to achromobactin biosynthesis (however, I do not know if the complete route is found in Sgm genome), which is a siderophore, possibly related to iron sequestration, together with the role of the ferritin. This could mean that, in the presence of heme-derived stress, heme uptake is inhibited and iron sequestration is activated to provide a source of iron, or alternatively to simply work as an iron chelator.

*Regarding these questions related to iron metabolism, we have added text in the discussion to address them. Specifically, we point out that SG1505 does have homology with the HmuS protein from Yersinia sp. which cleaves the protoporphyrin ring to release iron from heme in Yersinia pseudotuberculosis and that this protein is likely modulating heme degradation (lines 395-397). We also point out that tsetse’s genome encodes a heme oxygenase (lines 399-400). Finally, we also indicate that the S. glossinidius genome encodes a putative heme biosynthesis pathway, which is puzzling given the molecule’s abundance in blood and S. glossinidius’ ability to take it up (lines 89-90).*

*We did find that* *achromabactin biosynthesis and export proteins, but not import proteins, were ~2-fold upregulated in response to high heme. We chose not to mention this in the discussion because the idea that siderophores sequester iron is highly speculative.*

Why are there so many type III secretion apparatus proteins upregulated by heme?

*Contrary to the reviewer’s observation, we did not observe a significant upregulation in the expression of type III secretion apparatus genes following exposure to high heme. We observe that some relevant genes are insignificantly up or down regulated whereas others exhibit no change.*

This symbiont works as a biotin source for the insect? Heme inducing activation of biotin biosynthesis would be an interesting aspect of the mutualistic interaction between host and bacteria.

*We currently do not know if Sodalis serves as a biotin source for tsetse. We agree with the reviewer that the observed heme induction of Sodalis biotin biosynthesis genes (SG0902-SG0906) would be an interesting aspect of the mutualistic interaction between the fly and the bacterium, but that is beyond the scope of the work presented here. Interestingly, tsetse’s obligate symbiont, Wigglesworthia, also retains the capacity to synthesize biotin, although like with Sodalis, the functionality of these genes has not been determined.*

Reviewer #2: Although several genes are clearly related to a heme response, and the authors provide evidence that, at least for some selected transcripts, a similar expression profile is also found in the insect midgut, my feeling is that they are dealing with a profile that has a component of a general stress response, as they see a reduction of major ribosomal components (they are abundant among the heme-repressed genes). Does this downregulation of protein synthesis also occur in vivo? Also, several stress response proteins are upregulated.

*We appreciate the reviewer pointing out this possibility. We have added a paragraph (lines 405-422) to the discussion that describes how some of the differentially expressed Sgm genes we observed imply that the bacterium experiences a general stress response when exposed to high heme.*

The experimental design chosen was to use a heme concentration that is deleterious but not lethal. I can understand the rationale for that strategy. However: what are the “free” heme concentrations that are attained in the midgut in vivo? Is there an experimental evaluation of labile heme (an expression to describe the heme pool that is available to change from one binding partner to another)? As these data are probably lacking, at least, can the authors provide a description of the rate of blood protein degradation in Glossina? This might work as an indirect (but acceptable) evidence that labile heme  is being generated. The common pattern from published research is that heme released from hemoglobin is quickly converted into aggregates in the gut of mosquitoes, kissing bugs, and ticks, but, as long as I know, there are not such studies available for Glossina. So, I wonder if heme (labile) concentrations are in fact escalating to 100micromolar. 

*We have searched the literature and found no information about the heme concentration or protein degradation rate in tsetse’s midgut. We have been careful not to imply that our in vitro conditions for identifying heme inducted genes are reflective of the actual levels in vivo*.

Also, Sodalis is not the unique bacteria found in the Glossina midgut. The use of the antibiotic selects the mutant strain and allows comparison in a situation where other species are not present. As a simplifying experimental approach, this is ok but is a fact that needs to be recognized in the discussion.

*The reviewer is correct in that the use of antibiotics does select for mutant Sodalis strains. However, we did not use antibiotics when colonizing tsetse per os during our colonization assays. We only used antibiotics as a selection tool when plating and counting the number of mutant clones versus the number of parental clones in the gut of experimentally colonized flies. Both mutant and parental strains resided in tsetse’s gut along with wild-type cells, but the latter were selected out so as to count only the inoculated mutant or parent cells.*

Reviewer #1: Runyen-Janecky et al report on an RNAseq analysis of genes regulated by heme exposure in the gut endosymbiont of tsetse. They hypothesized that as part of the process of adaptation to become a commensal in the gut of an obligatory bloodfeeder, the Sodalis bacteria should be able to tolerate extreme heme concentrations. Indeed, their data show this is the case and that many genes are differentially regulated in the bacteria in response to heme. Of these, a subset were mutagenized and shown to reduce colonization of the gut, demonstrating their adaptive nature. Overall, this is a well put together, original study that makes an important contribution to the field of vector biology. I will add that the manuscript is also extremely well written, and should be accessible to the broad audience at PLoS NTD. I only have a few minor concerns and some suggestions for data presentation that I hope the authors will consider.

In Figure 4, the authors show the results of colonization experiments with Sodalis strains deficient in various genes that had responded to heme. There are no error bars on the graph, even though the methods and legend indicate there were replicates. From the legend, N=25 per group, with n=5 for each data point. So should be five data points per time period. The graph itself is also odd. The Y-axis is labelled "log2 CFU/gut (Sgm-mutant/Sgm-WT)”, and this is fine. However, the numerical labels are not log2 values, they are linear values but on a log2 scaled axis. This is a bit confusing, as it would be simpler to just number from 1-4 (and -1 to-4), since those are the log2 values.

*As the reviewer suggested we have added error bars to the data in the graph. Also, we have clarified the Y-axis label by indicating that we plotted fold-change in mutant CFU’s/gut relative to the parental strain. We feel this makes the data more easily interpretable by the reader without us having to override the software’s default numbering of the axis.*

While the authors include all of the expression data values in the supplement, there is no corresponding figure in the manuscript that summarizes these data. There are many acceptable ways of showing the data: 1) linear plot of expression heme vs no heme for all genes; 2) volcano plot showing log2 fold change vs corrected p-value; 3) log10 (heme x non-heme expression) vs log2 fold change. Any of these would help to demonstrate the strong asymmetric shift in expression that occurs upon treatment in a way that is essentially overlooked without such a summary.

*As the reviewer suggested we have made a volcano plot showing fold change vs -Log_10_ p-value as a visual summary of the RNA-seq data we generated in this study. This volcano plot is designated S1 Fig. in the revised manuscript.*

The summary in Fig 2 is not very effect in communicating categories. There are too many similar colors, and the fonts on the figure are too small to read. Recommend changing to a different format.

*We have remade the figure into a more legible format.*

Reviewer #2: Overall, a very interesting paper, the first to study how a symbiont from the intestinal microbiota of a blood-sucking insect deal with heme. It creates a straightforward experimental approach using an obligate symbiont and combines a transcriptome and mutational strategy.

*We are very appreciative of Dr. Oliveira’s positive comments about our work.*
